# A new species of *Raputia* (Rutaceae) from the Selva Central of Peru

**DOI:** 10.3897/phytokeys.89.20136

**Published:** 2017-11-03

**Authors:** Robin Fernandez-Hilario, Robert Arteaga

**Affiliations:** 1 Herbario Forestal MOL, Facultad de Ciencias Forestales, Universidad Nacional Agraria La Molina, Av. La Molina s/n, La Molina, Lima, Perú; 2 Departamento de Ecología, Museo de Historia Natural “Javier Prado”, Universidad Nacional Mayor de San Marcos, Av. Arenales 1256, Jesús María, Lima, Perú

**Keywords:** *Raputia*, Galipeinae, premontane forests, Neotropic, *Raputia*, Galipeinae, Bosques premontanos, Neotrópico

## Abstract

*Raputia codo-pozuzoensis* Rob. Fernandez & Arteaga, **sp. nov.** (Galipeinae, Rutaceae), a new species endemic to the Huanuco region, in the Selva Central of Peru, is described and illustrated here. The new species differs from other species of *Raputia* by the combination of 5–7-foliolate leaves (a new characteristic for the genus) and longer petioles. A key to the four Peruvian species of *Raputia* is presented.

## Abstract (es)

*Raputia codo-pozuzoensis* Rob. Fernandez & Arteaga, **sp. nov.** (Galipeinae, Rutaceae), una nueva especie endémica del departamento de Huánuco, en la Selva Central del Perú, es descrita e ilustrada aquí. La nueva especie difiere de las otras especies de *Raputia* por presentar hojas 5–7-folioladas (una nueva característica para el género) y peciolos largos. Una clave para las cuatro especies peruanas de *Raputia* es presentada.

## Introduction

Rutaceae Juss., in the order Sapindales (APG III, 2009; APG IV, 2016), is a family of mostly tropical and sub-tropical trees, shrubs, and aromatic herbs comprising approximately 2100 species in 154 genera (Kubitzki et al. 2011). In recent years, numerous phylogenetic studies have revealed that the seven sub-families initially proposed by Engler (1931) were paraphyletic (Chase et al. 1999, Scott et al. 2000, Groppo et al. 2008, Groppo et al. 2012, Morton and Telmer 2014), a revelation that has prompted recircumscription of intra-familial groups and new understanding of relationships among them. For example, at the subfamily level, Groppo et al. (2012) reduced the number of subfamilies to two, and Morton and Telmer (2014) to four; and at a more specific level, Bruniera et al. (2015), in the first study of relationships within subtribe Galipeinae (tribe Galipeeae, subfamily Rutoideae), where *Raputia* belongs, transferred all the species of *Almeidea* A. St.-Hil. to *Conchocarpus* J.C. Mikan and determined that the Galipeinae is apparently a monophyletic group.

*Raputia* and its type species *Raputia aromatica* were established by Aublet in 1775 based on collections from forest near the Orapu River in French Guiana. In the first comprehensive classifications of the Rutaceae, Engler (1874, 1931) recognized nine species in the genus, forming a group with heterogeneous characteristics.

Emmerich (1978) split this group and placed most of the species in three more homogenous genera (*Neoraputia* Emmerich, *Sigmatanthus* Huber ex Emmerich and *Raputiarana* Emmerich). Subsequently, Kallunki (1990) emended the description of *Raputia* and making three new combinations. Finally, the last published revision of the genus was made by Kallunki (1994), where she recognized 10 species. Currently, *Raputia* is found in the subtribe Galipeinae Kallunki [Angostura Alliance sensu Kubitzki et al. (2011)] along with 25 other genera, all restricted to the Neotropics, and characterized by tendencies toward zygomorphic flowers, a more or less tubular corolla, union of the filaments to a corolla tube, reduction in number of fertile stamens from five to two with the transformation of stamens into staminodes, modifications of anthers, loss of endosperm, a curved embryo and conduplicate and plicate cotyledons (Bruniera et al. 2015, Kallunki and Pirani 1997, Kubitzki et al. 2011). In addition, in most taxa with only two fertile stamens, the anthers are variously modified by basal or apical appendages or sterile basal portions above the point of attachment to the filament and, in some, the anthers or appendages are united (Kubitzki et al. 2011).

Brako and Zarucchi (1993), in the “*Catalogue of the Flowering Plants and Gymnosperms of Peru*”, listed three species known from that country: *Raputia ulei* (K. Krause) Kallunki, *Raputia heptaphylla* Pittier and *Raputia magnifica* Engler. In the revision of the genus, Kallunki (1994) cited no specimens of *Raputia ulei* from Peru. Instead, she identified the collection (Vásquez et al. *8909*) that was the basis for the report of that species from Peru (Brako and Zarucchi, 1993) as *Raputia simulans* Kallunki. Furthermore, she excluded *Raputia heptaphylla* and *Raputia magnifica* from the genus because they showed discordant characteristics and subsequently recognized them as *Raputariana heptaphylla* (Pittier) Kallunki (Jiménez 2014), and *Neoraputia magnifica* (Engl.) Emmerich ex Kallunki (Kallunki 2009), respectively. Additionally, Kallunki (1994) described *Raputia megalantha* Kallunki and transferred *Achuaria hirsuta* Gereau to *Raputia hirsuta* (Gereau) Kallunki, which with *Raputia simulans* Kallunki, are known from Peru (Vasquez and Rodriguez 2002; Ulloa Ulloa et al. 2004).

*Raputia* comprises a total of 11 Neotropical species, occurring from Venezuela and French Guiana to Amazonian Colombia, Peru and Brazil, principally from lowland areas, with shrubby or tree-like habit, opposite 1–3-foliolate leaves, circinate cauline monochasium, and pentamerous flowers with bilabiate corolla (Kallunki 1994; Kubitzki et al. 2011; Pirani 2005).

During fieldtrips to the premontane forests of Huanuco (Peru) in 2015, we collected an undescribed species of *Raputia* with 5–7-foliolate leaves, a new characteristic for the genus.

## Taxonomic treatment

### Raputia codo-pozuzoensis

Taxon classificationPlantaeSapindalesRutaceae

Rob. Fernandez & Arteaga sp. nov.

urn:lsid:ipni.org:names:60475533-2

Figure 1 , 2

#### Diagnosis.

*Raputiacodo-pozuzoensis differs from others species in this genus by its 5–7-foliolate leaves and longer petioles (8.5–12.5 cm long).*

#### Type.

**PERU. Huánuco**: Prov. Puerto Inca, Dist. Codo de Pozuzo, alrededores de toma de agua cerca al Río Pozuzo, 565 m, 9°40'57.76"S, 75°30'31.35"W, 01 Feb 2015 (fl.), *R. Fernandez, R. Arteaga & F. Meza 830* (holotype MOL - 2 sheets).

#### Description.

**Monopodial shrub** up to 2 m tall; stem cylindrical, 1–1.5 cm in diameter, lenticellate and finely ribbed, dark brown; the terminal buds, young twigs and petioles, and inflorescences pubescent, the hairs short and antrorse. **Terminal twigs** circular in transverse section, 4–7 mm in diameter, beige-colored when dry, lenticellate; terminal buds ferruginous, stipules absent. **Leaves** palmately compound, 5–7-foliolate, opposite or verticillate; petiole cylindrical, 8.5–12.5 cm long, 2–3 mm wide; petiolule absent; leaflet blades elliptical, acuminate at apex, decurrent at base, entire at margin, discolorous, sub-chartaceous, the venation brochidodromus, the surface pellucid-punctate, the upper and lower surface glabrous, midrib pubescent beneath, the hairs short and antrorse; central leaflet (21-) 25–36 cm long, 3.5–6 cm wide, the secondary veins (18-) 21–29; lateral leaflet progressively smaller, the basal ones (7.5-) 11–18 cm long, 1.7–3.5 cm wide, the secondary veins 10–16. **Inflorescence** cauline, of 1–3 monochasia, with 6–14 flowers, 1.8–3 cm long including a peduncle 2–5 mm long. **Flowers** bisexual, zygomorphic, pentamerous; pedicel 1.5–2 mm long; flower buds slightly curved. **Calyx** 4–4.5 mm long, 3.5 mm wide at base of lobes, glabrous or pubescent; sepals fused at the base, 5-lobed, the lobes quincuncial, ovate, acute to obtuse at apex, 2 mm long, ciliate, pellucid-punctate. **Corolla** tubular, unequally 5-lobed, 12–17.5 mm long, bilabiate at anthesis, glabrous in the external surface, sparsely pubescent in the inner base of the tube, woolly in the inner part of the throat, the trichomes ca. 1.2 mm long; the tube white to yellowish, 2–6 mm long to the sinuses of the innermost lobe (inferior lip), 6–9 mm long to the sinuses of the other 4 lobes (superior lip), recurved superior lip; the lobes green, imbricate, oblong, rounded at apex, the inner lobe 10–12 mm long, 3.5–5 mm wide, the other 4 lobes 5–6 mm long, 3.5–4.5 mm wide, pellucid-punctate. **Androecium** of 2 fertile stamens and 3 staminodia, white-colored; filaments of fertile stamens flanking the inner lobe, adherent from the base to the throat of the corolla tube, the free portion above the throat ca. 2 mm long; staminodia adherent from the base to the throat of the corolla tube, the free portion linear above the throat 9–11 mm long, alternate with the other four corolla lobes; filaments of fertile stamens and staminodia glabrous at the base and apex, only bearded at the throat of the corolla, filaments and back of anthers pellucid-punctate; anthers lanceolate, laterally coherent, basifixed, ca. 5.5 mm long, 1.5 mm wide, glabrous, the appendages flattened, ca. 1.5 mm long, 1 mm wide, glabrous. **Gynoecium**, ovary of 5 carpels united at the base and by single style, 1.5 mm in diameter, 1 mm high, furrowed, orange-colored; the style 10–11 mm long, slightly curved, glabrous, pellucid-punctate; the stigma 1 mm in diameter, slightly 5-lobed; disc cupular enveloping the ovary, 2.5 mm in diameter, 1.5 mm high, margin 5-lobed, cream-colored, glabrous. **Fruit** not seen.

**Figure 1.**
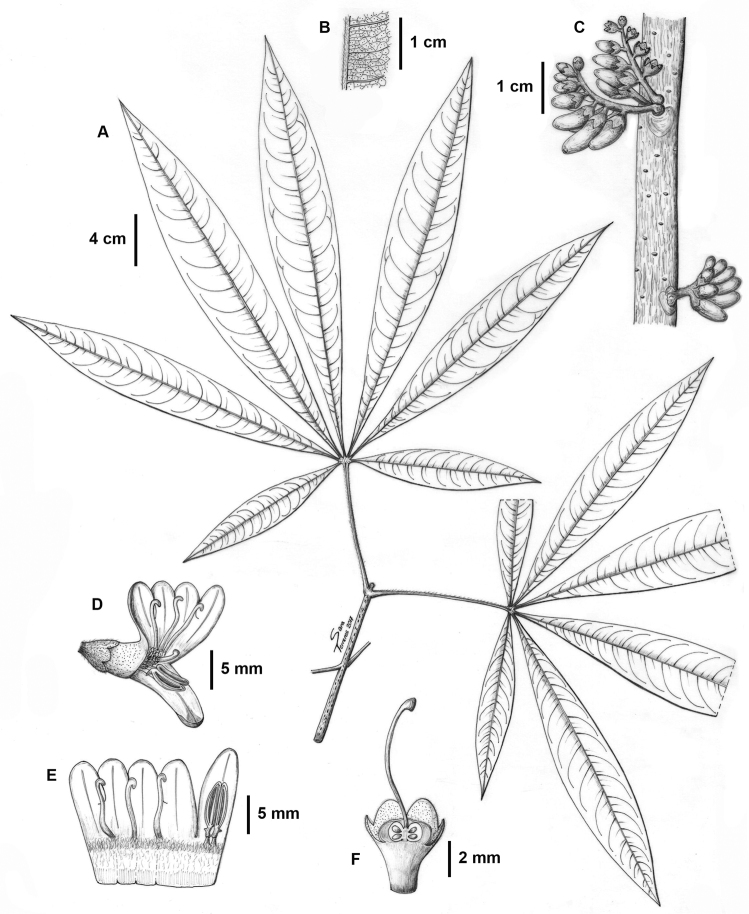
*Raputia codo-pozuzoensis*. **A** Terminal twig **B** Midrib beneath and lower leaf surface **C** Inflorescences **D** Flower **E** Corolla opened showing two fertile stamens and staminodes **F** Longitudinal section of calyx, disc cupular, ovary and style. From *R. Fernandez* et al. *830* (MOL). Drawing by Sara Terreros.

**Figure 2.**
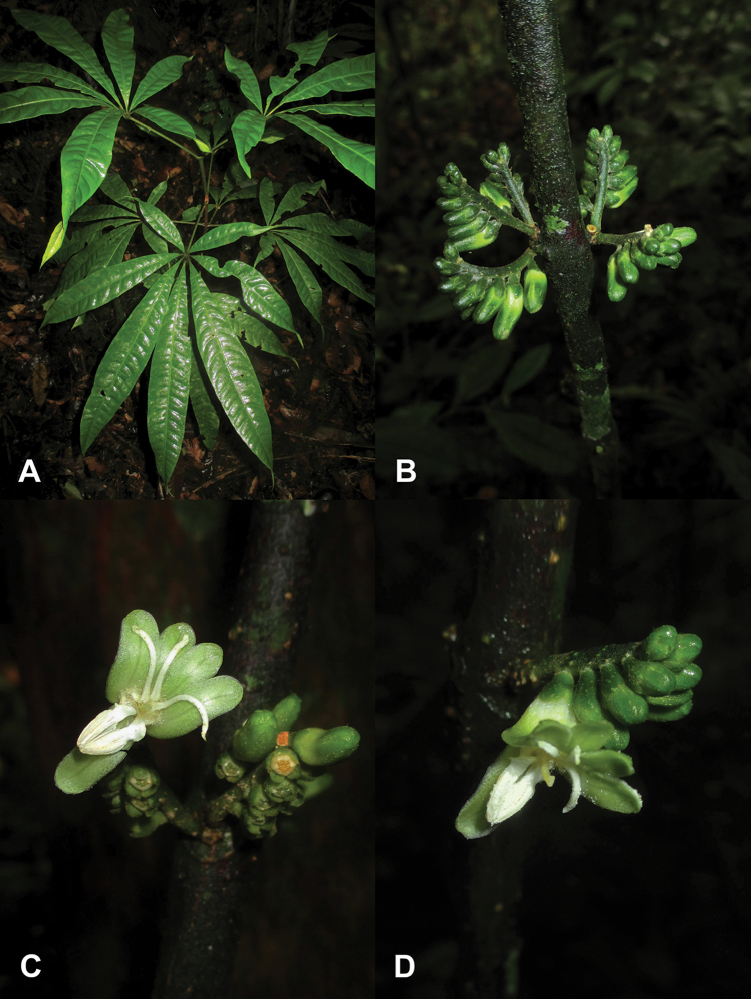
*Raputia codo-pozuzoensis*. **A** Habit **B** Inflorescence (*R. Fernandez & R. Arteaga 1079*; MOL) **C, D** Flowers (*R. Fernandez* et al. *830*; MOL). Photos by Robin Fernandez.

#### Distribution and habitat.

*Raputia codo-pozuzoensis* is endemic to humid premontane forest in central Peru, between 565–589 m.a.s.l., growing in zones with shallow to steep slopes in a loamy-silty soil. The only known population of this species occurs in the understory of a forest of tree species, such as: *Chrysophyllum sanguinolentum* (Pierre) Baehni, *Helicostylis scabra* (J.F. Macbr.) C.C. Berg, *Hevea guianensis* Aubl., *Iryanthera hostmannii* (Benth.) Warb., *Mabea speciosa* Müll. Arg., *Macrolobium gracile* Spruce ex Benth., *Theobroma subincanum* Mart. and *Virola pavonis* (A. DC.) A.C. Sm.

**Figure 3.**
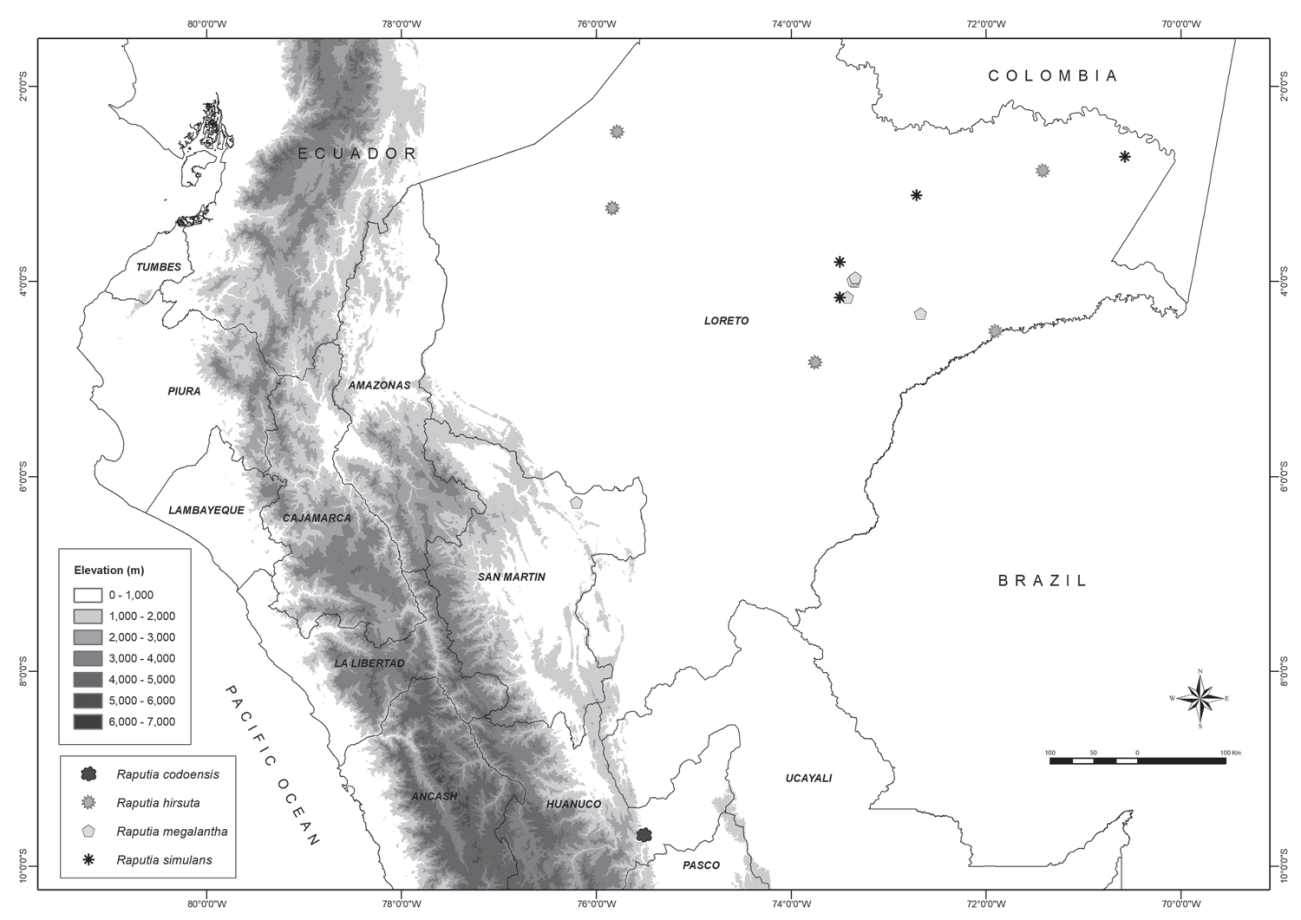
Distribution of *Raputia* species in Peru.

#### Etymology.

The specific epithet refers to the Codo de Pozuzo district, the only place where the specimens were found and collected.

#### Phenology.

Flowering take place from December to February.

#### Conservation state.

We collected individuals of *Raputia codo-pozuzoensis* in areas of slightly disturbed forest, and we observed individuals sprouting after being cut for the establishment of “*trochas*” (pathways). We counted 20 individuals in an area of 0.5 ha. Thus, we assume that human activities are not affecting seriously the wild populations of this species. Nonetheless, in our inventories at other sites in Puerto Inca Province and surrounding areas, we and our collaborators have not observed other populations of this new species. Additionally, the extent of occurrence estimated of this species has been decreasing over the last years by deforestation and only remaining less than 100 km^2^ of the original forest cover. Therefore, under the guidelines of UICN (2012), we assign this species to the category Critically Endangered [CR (B1a+bi)].

#### Additional specimens examined.

**PERU. Huánuco**: Prov. Puerto Inca, Dist. Codo de Pozuzo, alrededores de toma de agua cerca al Río Pozuzo, 589 m, 9°40'56.72"S, 75°30'30.85"W, 28 Dec 2015 (fl.), *R. Fernandez & R. Arteaga 1079* (MOL), *R. Fernandez & R. Arteaga 1080* (USM), *R. Fernandez & R. Arteaga 1081* (HOXA).

#### Discussion.

According to the morphologic analyses of Kubitzki et al. (2011), *Raputia* belongs to a natural group along with the following genera: *Apocaulon* R.S. Cowan, *Decagonocarpus* Engl., *Ertela* Adans., *Lubaria* Pittier, *Ravenia* Vell. and *Raveniopsis* Gleason, characterized by their opposite leaves (alternate in *Apocaulon*), overlapping sepals, connate petals, basally appendaged (exc. in *Ertela*) and often laterally coherent anthers, reticulate pollen grains, apocarpous gynoecia, and conduplicate, bilobed cotyledons. Among this group, the two herbaceous genera, *Apocaulon* and *Ertela*, stand out and differ noticeably from the others. The former, by its alternate leaves and anthers coherent by their adaxial surfaces, and the later by its strongly unequal sepals, with the two outer much larger and concealing the corolla, and the anthers lacking basal appendages. Previously, Kallunki (1994) recognized that *Raputia* was related with the following genera: *Decagonocarpus*, *Lubaria*, *Ravenia* and *Raveniopsis*, forming a group characterized by opposite leaves, a quincuncial calyx (of which the margins are conspicuously overlapping at anthesis), and conduplicate, bilobed cotyledons. Kallunki (1994) differentiated *Raputia* from these other four genera by the presence of cauline inflorescences and the leathery testa (vs. terminal inflorescences and crustaceous testa). Even though we did not register neither the seeds nor the fruits of *Raputia codo-pozuzoensis*, the combination of characteristics such as cauline inflorescences, the petals connate, forming a bilabiate corolla with a short tube, and anthers laterally connate, with basal appendages, allow us to locate this new species in the *Raputia* genus. In Table 1 we display the different characteristics of genera related to *Raputia*, according to Kubitzki et al. (2011).

**Table 1.** Comparison of *Raputia* with the morphologically most similar genera. Based on Albuquerque (1976), Kallunki (1994, 2005), Pirani (2005) and Kubitzki et al. (2011).

Character	*Apocaulon*	*Decagonocarpus*	*Ertela*	*Lubaria*	*Raputia*	*Ravenia*	*Raveniopsis*
Habit	Herbs	Shrubs or trees	Herbs, sometimes suffruticose	Trees	Shrubs or trees	Shrubs or trees	Shrubs or trees
Leaf characters	Leaves alternate, congested, often appearing basal, 3-foliolate	Leaves opposite, simple	Leaves usually opposite on lower part of stem, sometimes alternate or subopposite on upper part, 3-foliolate	Leaves opposite, simple	Leaves opposite, 1–7-foliolate	Leaves opposite or appearing alternate due to reduction of one of two opposite leaves, simple or 3-foliolate	Leaves opposite (in *Raveniopsis steyermarkii* R.S. Cowan some alternate), 1–3-foliolate
Inflorescence position	Axillary	Terminal	Terminal but sometimes appearing axillary	Terminal	Cauline (rarely axillary)	Terminal	Axillary or terminal
Inflorescence type	Dichasium	Monochasium	Dichasium	Dichasium	Monochasium	Dichasium, monochasium, or 1 or 2 flowers	Dichasium, monochasium, a congested capitulate thyrse, or a solitary flower
Calyx aestivation	Unknown	Quincuncial	Quincuncial	Quincuncial	Quincuncial	Quincuncial	Quincuncial
Calyx features	Sepals 5, strongly unequal, shortly coherent	Cupular, ± equally 5-lobed	Sepals 5, free, strongly unequal, the 2 outer much larger and concealing the corolla	Sepals 5, free, the 2 outer larger	Sepals 5, connate at very base, ± unequal	Sepals 5, the 2 outer larger than inner	Sepals 5, free or very shortly connate, usually unequal
Corolla aestivation	(4)5 imbricate lobes	Induplicate-valvate	Imbricate	Imbricate	Imbricate	Imbricate	Imbricate
Corolla features	Petals 5, connate, the tube curved, the 2 lobes opposite the lobe flanked by the 2 stamens joined for a slightly longer distance and forming a bilobed lip	Petals 5, connate into a long, slightly curved tube with recurved to spreading lobes, the tube longer than the lobes	Petals 5, connate, forming a bilabiate corolla with a short tube, one lip formed by the innermost petal, the other lip 4-lobed	Petals 5, the adaxial, innermost one free, the others connate into a 4-lobed lip	Petals 5, connate, forming a bilabiate corolla with a short tube, one lip formed by the innermost petal, the other lip forming a 4-lobed, recurved lip	Petals 5, connate to the middle or more, forming a bilabiate corolla	Petals 5, connate, forming a slightly zygomorphic to markedly bilabiate corolla, the corolla tube cylindric, slightly curved, longer than or equal to the lobes
Anthers	Coherent by their adaxial surfaces, with basal appendages	Laterally coherent, with basal appendages	Laterally coherent in lower half, lacking basal appendages	Laterally coherent, with basal appendages	Laterally connate, with basal appendages	Laterally coherent or not, with or without basal appendages	Sometimes laterally coherent, with basal appendages
Testa	Crustaceous	Crustaceous	Crustaceous	Crustaceous	Leathery	Crustaceous	Crustaceous
Cotyledons	Conduplicate, emarginate at apex	Conduplicate, bilobed at apex	Conduplicate, bilobed at apex	Conduplicate, bilobed at apex	Conduplicate, thick, stiff, bilobed at apex	Conduplicate, fleshy, bilobed at apex	Conduplicate, bilobed at apex (or rarely incumbent and rounded at apex)
Number of species	1	2	2	1	12	11	19

*Raputia codo-pozuzoensis* is easily distiguished from all other species of the genus by its 5–7-foliolate palmately compound leaves. The other three species found in Peru show unifoliolate (*Raputia simulans*) or three-foliolate leaves (*R. hirsuta* and *R. megalantha*). *Raputia codo-pozuzoensis* differs further from *R. simulans* by its much shorter inflorescences 1.8–3 cm long (vs. 19.5–26.5 cm) and from *Raputia hirsuta* by its short and antrorse hairs (vs. hirsute) on stems, leaves, and inflorescences.

Like *Raputia megalantha* and *Raputia maroana* (R.S. Cowan) Kallunki, *Raputia codo-pozuzoensis* possesses inflorescences shorter than 6 cm and terminal leaflets longer than 20 cm. *Raputia codo-pozuzoensis* differs, however, from *R. megalantha* by its petioles 8.5–12.5 cm long (vs. 0.8–3.3 cm) and its corollas 12–17.5 mm long (vs. 30 mm). Although *Raputia codo-pozuzoensis* shares with *R. maroana* petioles and corollas of similar lengths, it differs from the latter by its 5–7-foliolate (vs. 3-foliolate) leaves and filaments ca. 2 mm (vs. 11–12 mm) long. In addition, *Raputia codo-pozuzoensis* is restricted to premontane forest in southwestern Amazonia (Huanuco, Peru), whereas *R. megalantha* and *R. maroana* are distributed in lowland forests in northwestern Amazonia (Brazil, Peru, and Venezuela; Kallunki, 1994).

### Key to the species of *Raputia* in Peru

**Table:** 

1	5–7-foliolate leaves, petioles longer than 8 cm	***R. codo-pozuzoensis***
–	1–3-foliolate leaves, petioles shorter than 4 cm	**2**
2	Leaves 1-foliolate; inflorescences longer than 16 cm; flowers more than 20	***R. simulans***
–	Leaves 3-foliolate; inflorescences shorter than 7 cm; flowers fewer than 15	**3**
3	Central leaflet 8–30 cm long; inflorescences and petioles hirsute	***R. hirsuta***
–	Central leaflet 50–71 cm long; inflorescences and petioles strigulose	***R. megalantha***

### Additional Peruvian specimens of other species

***Raputia hirsuta.* PERU. Loreto.** Prov. Coronel Portillo, Padre Abad, granja del sr. Barrera, NE de la chacra de César Vela (Aguaytia), 17 Oct 1972, *V. Schunke 5396* (F, MO, NY); Prov. Loreto, Río Tigre, San Jacinto, Campamento de Occidental Petroleum, 175–205 m, 02°28'S, 75°47'W, 08 Jun 1993, *H. Beltrán & R. Foster 435* (F, USM); Prov. Loreto, Dist. Loreto, Pampa Hermosa and vicinity, 3°15'S, 75°50'W, 03–20 Dec 1985, *W. Lewis* et al. *10328* (F, MO, USM), 04–09 Jun 1986, *W. Lewis* et al. *10729* (MO, USM), 04–09 Jun 1986, *W. Lewis* et al. *10782* (MO, USM); Prov. Loreto, Campamento Petrolero San Jacinto, Rio Tigre, 2°15'S, 75°50'W, 16 Sep 1979, *C. Díaz & N. Jaramillo 1454* (MO); Prov. Mariscal Ramón Castilla, Margen izquierda del Río Yavari, entre Colonia Angamos y Lago Preto, 4°30'53"S, 71°54'2.77"W, 10 Apr 2003, *H. Beltrán* et al. *5743* (AMAZ, USM); Prov. Mariscal Ramón Castilla, Alto Río Yaguas, tributario del Río Putumayo, aprox. 80 km NE de Pebas, 140 m, 02°51'53.5"S, 71°24'54.1"W, 07 Aug 2003, *M. Ríos* et al. *537* (F); Prov. Maynas, Río Blanco, a 3 horas (Jonhson 40 Hp) desde Tamshiyacu, 130 m, 15 Mar 1978, *C. Díaz* et al. *145* (MO); Prov. Requena, Sapuena, Jenaro Herrera, 170 m, 4°50'S, 73°45'W, 12 Nov 1987, *R. Vásquez* et al. *9983* (MO, USM).

***Raputia megalantha.* PERU. Loreto.** Prov. Maynas, Mishuyacu, near Iquitos, 100 m, Oct-Nov 1929, *G. Klug 544* (F, NY, US); Prov. Maynas, Estación Biológica Rio Blanco, 04°20'S, 72°45'W, 16 Sep 1985, *R. Vásquez* et al. *6743* (MO, NY); Prov. Maynas, Dist. Allpahuayo, Estación Experimental del IIAP, 04°00'15"S, 73°25'48"W, 30 May 1990, *R. Vásquez* et al. *13786* (MO, USM); Prov. Maynas, Dist. Iquitos, carretera del caserío de Puerto Almendras, 26 Jun 1984, *M. Rimachi 7529* (US); Prov. Maynas, Dist. Iquitos, San Juan, km 31.5 carrera Iquitos-Nauta, 160 m, 3°59'34.7"S, 73°26'42.5"W, 08 Sep 2002, *M. Flores* et al. *1690* (AMAZ, MO, USM); Prov. Maynas, Dist. Iquitos, Allapahuayo, Estación del IIAP, 150–180 m, 04°10'S, 73°30'W, 18 Jun 1991, *R. Vásquez 16810* (AMAZ, MO, NY); Prov. Maynas, Dist. Iquitos, Allpahuayo, Estación Experimental del IIAP, 03°58'16"S, 73°25'08"W, 11 Oct 1990, *R. Vásquez & N. Jaramillo 14495* (MO); Prov. Maynas, Dist. San Juan, Reserva Nacional Allpahuayo-Mishana, 128 m, 03°58'02"S, 73°25'08"W, 19 Nov 2008, *R. Vásquez* et al. *35060* (HOXA). **San Martín.** Prov. Lamas, Caserio Bonilla, trail to E of road, Km 75 of Tarapoto-Yurimaguas road, 250 m, 6°16'S, 76°17'W, 20 Apr 1986, *S. Knapp & J. Mallet 7138* (USM, MO, NY); Prov. Lamas, Santa Rosa de Davidcillo, trail to E of road to Tioyacu, 200 m, 6°16'S, 76°17'W, 22–23 Apr 1986, *S. Knapp & J. Mallet 7162* (USM, MO, NY); Prov. Lamas, Santa Rosa de Davidcillo, 220 m, 6°15'S, 76°17'W, 21 Aug 1986, *S. Knapp 8109* (MO, USM).

***Raputia simulans.* PERU. Loreto.** Prov. Mariscal Ramón Castilla, cabeceras del Río Apayacu, Noroeste de Pebas, 150 m, 03°07'00"S, 72°42'43"W, 17 Aug 2003, *M. Ríos* et al. *680* (F, NY); Prov. Maynas, Dist. Iquitos, Quebrada Aucaya, trocha de la cooperativa, 11 Aug 1973, *S. McDaniel & M. Rimachi 17701* (MO, NY); Prov. Maynas, Dist. Iquitos, Nina rumi-Rio Nanay, 122 m, 03°48'S, 73°25'W, 05 Mar 1987, *R. Vásquez* et al. *8909* (MO, NY); Prov. Maynas, Dist. Iquitos, Estación Experimental del IIAP, 04 Nov 1990, *R. Vásquez & N. Jaramillo 14560* (MO, NY), 150–180 m, 04°10'S, 73°30'W, Nov 1990, *R. Vásquez & N. Jaramillo 14626* (MO), 24 May 1991, *R. Vásquez & N. Jaramillo 16426* (MO); Prov. Maynas, Dist. Iquitos, Allpahuayo, 150 m, 04°10'S, 73°30'W, 19 Mar 1992, *R. Vásquez 17727* (MO); Prov. Maynas, Dist. Iquitos, carretera Iquitos-Nauta, km 28, trocha del Fundo Pichiri, 150 m, 23 Jul 1997, *M. Rimachi 12005* (USM); Prov. Maynas, Dist. Putumayo, NE de Iquitos y Pebas, en la esquina del trapezoide de Colombia, 80 m, 02°43'15.9"S, 70°34'30.6"W, 28 Oct 2010, *I. Huamantupa* et al. *14752* (F).

## Supplementary Material

XML Treatment for Raputia codo-pozuzoensis
